# *SCN1B*‐linked early infantile developmental and epileptic encephalopathy

**DOI:** 10.1002/acn3.50921

**Published:** 2019-11-11

**Authors:** Alec Aeby, Claudine Sculier, Alexandra A. Bouza, Brandon Askar, Damien Lederer, Anne‐Sofie Schoonjans, Marc Vander Ghinst, Berten Ceulemans, James Offord, Luis F. Lopez‐Santiago, Lori L. Isom

**Affiliations:** ^1^ Pediatric Neurology Queen Fabiola Children Hospital ULB Brussels Belgium; ^2^ Pediatric Neurology ULB‐Hôpital Erasme Brussels Belgium; ^3^ Department of Pharmacology University of Michigan Medical School Ann Arbor MI 48109; ^4^ Centre for Human Genetics IPG Charleroi Belgium; ^5^ Pediatric Neurology Universiteit Antwerpen (UA) Antwerp Belgium; ^6^ ENT Department ULB‐Hôpital Erasme Université libre de Bruxelles (ULB) Brussels Belgium; ^7^ Laboratoire de Cartographie fonctionnelle du Cerveau UNI – ULB Neuroscience Institute Université libre de Bruxelles (ULB) Brussels Belgium

## Abstract

**Objective:**

Patients with Early Infantile Epileptic Encephalopathy (EIEE) 52 have inherited, homozygous variants in the gene *SCN1B*, encoding the voltage‐gated sodium channel (VGSC) *β*1 and *β*1B non‐pore‐forming subunits.

**Methods:**

Here, we describe the detailed electroclinical features of a biallelic *SCN1B* patient with a previously unreported variant, p.Arg85Cys.

**Results:**

The female proband showed hypotonia from birth, multifocal myoclonus at 2.5 months, then focal seizures and myoclonic status epilepticus (SE) at 3 months, triggered by fever. Auditory brainstem response (ABR) showed bilateral hearing loss. Epilepsy was refractory and the patient had virtually no development. Administration of fenfluramine resulted in a significant reduction in seizure frequency and resolution of SE episodes that persisted after a 2‐year follow‐up. The patient phenotype is more compatible with early infantile developmental and epileptic encephalopathy (DEE) than with typical Dravet syndrome (DS), as previously diagnosed for other patients with homozygous *SCN1B* variants. Biochemical and electrophysiological analyses of the *SCN1B* variant expressed in heterologous cells showed cell surface expression of the mutant *β*1 subunit, similar to wild‐type (WT), but with loss of normal *β*1‐mediated modification of human Na_v_1.1‐generated sodium current, suggesting that *SCN1B*‐p.Arg85Cys is a loss‐of‐function (LOF) variant.

**Interpretation:**

Importantly, a review of the literature in light of our results suggests that the term, early infantile developmental and epileptic encephalopathy, is more appropriate than either EIEE or DS to describe biallelic *SCN1B* patients.

## Introduction

Voltage‐gated sodium channels (VGSCs) are responsible for the rising phase and propagation of action potentials in excitable cells.^1^ VGSCs were purified as heterotrimeric complexes of *α* and *β* subunits from mammalian brain.^2^ This work showed that a central *α* subunit forms the ion‐conducting pore and is associated with two different *β* subunits.^3^ Originally characterized as “auxiliary,” *β* subunits are now known to be dynamic, multifunctional molecules that engage in diverse and essential roles in multiple tissues.^4^, ^5^ Their ability to participate in both conducting and nonconducting roles makes VGSC *β* subunits unique among voltage‐gated ion channel subunits. The breadth of *β* subunit function hinges on the key structural motif common to all members of this family of proteins: an immunoglobulin (Ig) loop enabling them to function as cell adhesion molecules (CAMs).^4^, ^5^, ^6^ CAM‐mediated adhesive functions are critical to brain development, including the processes of neurite outgrowth, axon pathfinding, fasciculation, and cell migration.^4^, ^5^ Integrity of the Ig loop is also critical for *β*1‐mediated VGSC modulation in vivo,^7^ making this domain multi‐functional. In their roles as ion channel modulators, not only of VGSCs but also of voltage‐gated K^+^ channels,^8^, ^9^, ^10^, ^11^ *β* subunits make important contributions to the regulation of neuronal firing. As substrates for sequential cleavage by *β*‐ (BACE) and *γ*‐secretases, *β* subunits contribute to the regulation of VGSC *α* subunit gene expression.^12^, ^13^ Taking all of these roles into consideration, it is not surprising that variants in the genes encoding VGSC *β* subunits are linked to pathophysiology.

Patients with Early Infantile Epileptic Encephalopathy (EIEE) 52 (OMIM 617350) have inherited, homozygous variants in the gene *SCN1B*, encoding the VGSC *β*1 and *β*1B non‐pore‐forming subunits.^5^ Inherited, heterozygous *SCN1B* variants have been linked to generalized epilepsy with febrile seizures (FS) plus, temporal lobe epilepsy (TLE), and cardiac arrhythmias, including Brugada syndrome and atrial fibrillation.^5^, ^14^ Homozygous *SCN1B* variants have been reported in seven epilepsy patients to date, with clinical descriptions suggestive of Dravet syndrome (DS).^15^, ^16^, ^17^ *Scn1b* null mice have a phenotype that is similar to DS.^18^, ^19^ Thus, this small patient cohort was presumed to represent a subset of DS. Here, we describe a 4‐year electroclinical follow‐up of an eighth reported *SCN1B* patient with a previously unreported variant, c.253C > T (p.Arg85Cys), located in the extracellular Ig loop domain. The electroclinical profile suggests a diagnosis of early infantile developmental and epileptic encephalopathy (DEE), a severe neurodevelopmental disorder often beginning in infancy that is characterized by intractable seizures and pronounced developmental impairment, a more severe form of epilepsy and developmental delay than DS. Electrophysiological analysis of the variant shows *β*1 subunit loss‐of‐function (LOF) in spite of normal cell surface expression of the protein. These data are similar to previous work with the *SCN1B*‐p.Cys121Trp variant, also located in the Ig loop domain, showing normal cell surface expression, but LOF in terms of sodium current (I_Na_) modulation^7^, ^20^ and for which a homozygous mouse model shows a phenotype that is similar to *Scn1b* null mice.^21^ Importantly, our results provide new information about autosomal recessive inheritance in epilepsy (reviewed in 22) and suggest reconsideration of the linkage of *SCN1B* variants to early infantile DEE rather than to EIEE or DS.

## Results

### Case study

**Figure 1:**
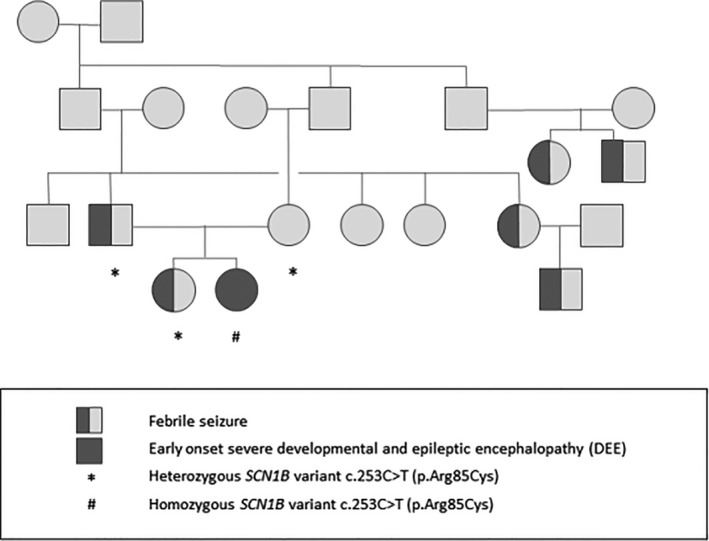
Pedigree of the family.

The female proband was born from consanguineous parents, first cousins of Belgian origin. The familial pedigree was highly suggestive of GEFS+ (Fig. 1). The father and his sister presented with febrile seizures (FS) in childhood. Three cousins of the parents had a non‐detailed history of FS or epilepsy.

**Figure 2:**
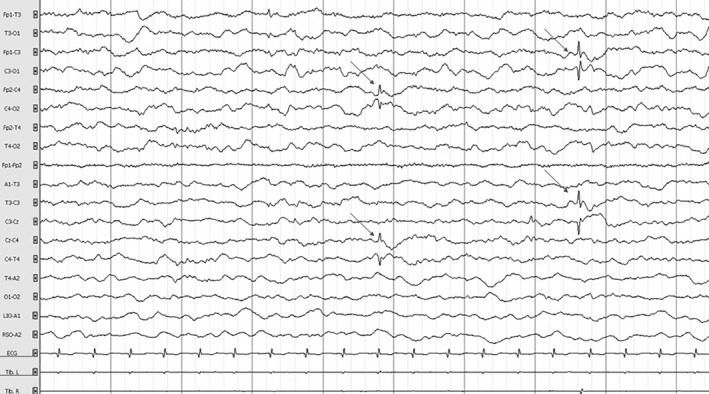
Electroencephalogram of the patient at 2.5 months in awake state showing a normal background with isolated, bilateral slow amplitude central spikes without clinical manifestations (arrows). Time constant: 10 sec. Amplitude: 100 µV/cm. High band filter: 0.3 Hz. Low band filter: 70 Hz.

**Figure 3:**
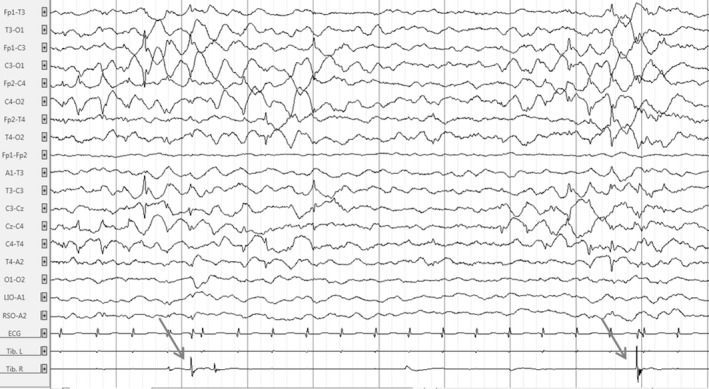
Electroencephalogram of the patient at 2.5 months in awake state showing clusters of bilateral high‐amplitude central spikes followed by high‐amplitude slow waves and erratic focal right myoclonus (arrows). Time constant: 10 sec. Amplitude: 100 µV/cm. High band filter: 0.3 Hz. Low band filter: 70 Hz.

**Figure 4:**
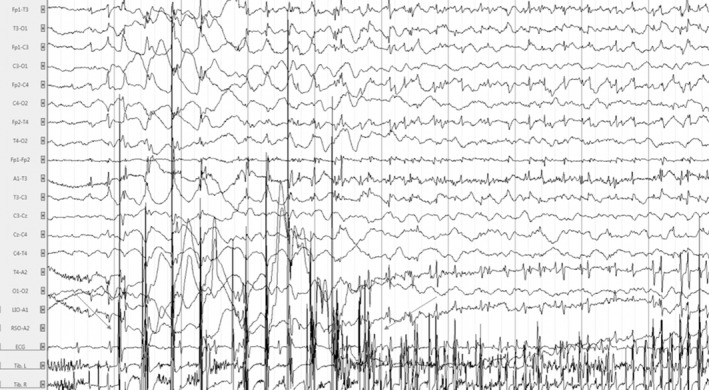
Electroencephalogram of the patient at 2.5 months in awake state showing rhythmic bilateral central high‐voltage spikes accompanied by a cluster of high‐voltage myoclonus (*n* = 10, arrow to arrow) followed by a focal to bilateral clonic seizure with alteration of consciousness lasting 80 sec. Time constant: 10 sec. Amplitude: 300 µV/cm. High band filter: 0.3 Hz. Low band filter: 70 Hz.

After an uncomplicated pregnancy, a global hypotonia was noted from birth. At 2 months of age, the proband was unable to hold her head, had no visual interactions, and began to present frequent erratic myoclonus triggered by transition phases of sleep, fever, and hot bath. Long‐term (24 h) video electroencephalogram (EEG) monitoring at that time revealed frequent bilateral central spikes (Fig. 2). Spikes were isolated or occurred in bursts lasting a few seconds followed by high‐voltage slow waves followed sometimes by unilateral or bilateral myoclonus (Fig. 3). Myoclonus was also observed without simultaneous spikes. Less frequently, there were bilateral temporal spikes. One cluster of myoclonus was followed by a focal to bilateral clonic seizure (Fig. 4), and thousands of epileptic and non‐epileptic myoclonus (Fig. 3) were captured during this first long‐term video EEG. Despite the frequent central and temporal spikes, structure of sleep stages was overall preserved with the presence of sleep spindles.

Despite the levetiracetam (50 mg/kg/d) treatment, erratic bursts of myoclonus persisted. The proband experienced a first fever‐induced myoclonic status epilepticus (SE) at 3 months, which lasted for 30 min. Several episodes of focal to bilateral tonic‐clonic seizures followed. Central spikes and waves with some generalized spikes were still observed on EEG. Addition of valproate (30 mg/kg/d) with clobazam (0.5 mg/kg/d) resulted in a slight decrease in both spikes and seizures. At 7 months, introduction of the ketogenic diet and topiramate (5 mg/kg/d) decreased myoclonus frequency and intensity. The proband became slightly more interactive with social smiling but with persistent, severe global hypotonia.

**Figure 5:**
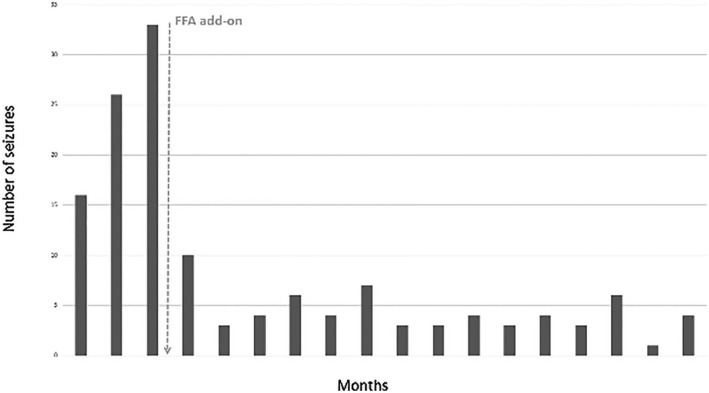
Number of seizures lasting more than 1 minute before and after fenfluramine (FFA) add‐on.

Eight myoclonic or hemiclonic febrile SE occurred within the first 2 years of life. At 24 months, EEG was unchanged, with thousands of myoclonus isolated or in bursts during the awake state or during sleep that often led to arousal. The epilepsy remained refractory, with developmental plateauing. The proband was treated at that time with a combination of valproic acid (30 mg/kg/d), topiramate (5 mg/kg/d), clobazam (0.5 mg/kg/d), and ketogenic diet. Fenfluramine (FFA) (0.6 mg/kg/d) was started as an add‐on at 28 months. No further SE has occurred since that time, despite weaning of the ketogenic diet at age 3. Moreover, a significant reduction in seizure frequency was observed (Fig. 5). The 24‐h video EEG performed 1 year after addition of FFA revealed no myoclonus during the awake state, even when the background EEG remained unchanged. During sleep, several epileptic and non‐epileptic myoclonus were recorded; however, they were less severe than observed prior to FFA administration and did not lead to arousal of the child. Taken together, these observations suggest that FFA was effective in reducing epileptic severity. Nevertheless, her motor and cognitive development remained severely impaired with little improvement since the beginning of epilepsy: interaction is present with social smiling and vocalization, but the child is still unable to hold her head at 5 years, with a severe global hypotonia.

**Figure 6:**
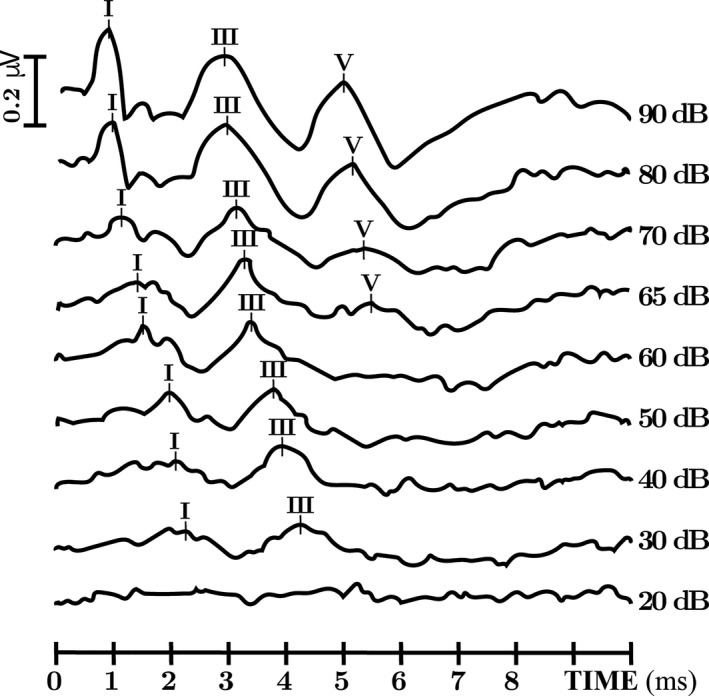
Auditory brainstem response obtained at various intensities decreasing from 90 to 20 dB alternatively in condensation and rarefaction polarity. ABR show, when stimulation intensity decreases, an early loss of the wave V, while wave I/III remains, and a prolonged I–V interpeak latencies. (For illustration purpose, only the right side is shown).

The work‐up showed normal metabolic analysis and normal brain MRI. Auditory brainstem response (ABR) indicated brainstem involvement, since wave V vanished bilaterally at 50 dB, while waves I and III were still identified at 30 dB. Evoked otoacoustic emissions were preserved, and both the behavioral audiometry and the auditory steady‐state responses estimated a bilateral hearing loss with a hearing threshold around 50 dB (Fig. 6). Electrocardiogram, holter ECG, and cardiac echography were normal (data not shown). A gene panel testing of DNA amplified by multiplex PCR (Ampliseq^®^) and re‐sequenced by next‐generation sequencing on Ion PGM® found a homozygous *SCN1B* variant c.253C > T (p.Arg85Cys) in the proband. Both parents and her older sister were found to be heterozygous carriers (Fig. 1). Other family members were not tested for the presence of the variant.

### *β*1‐p.Arg85Cys is expressed at the cell surface

**Figure 7:**
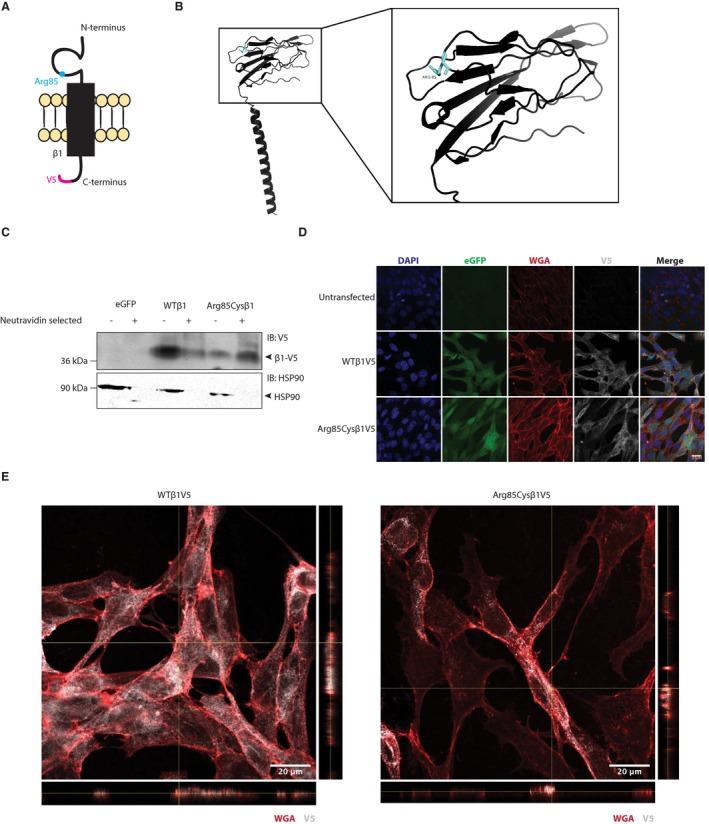
*β*1‐p.Arg85Cys localizes to the plasma membrane. (A) Cartoon diagram of *β*1‐p.Arg85Cys. (B) Crystal structure of WT*β*1 (PDB: 6AGF).^23^ The residue, Arg85, is shown in cyan. Right: 20 angstrom area showing detail of the Ig domain. (C) Cell surface biotinylation shows that *β*1‐p.Arg85Cys can localize to the plasma membrane, similar to WT (representative of four independent experiments). (D) *β*1 WT and *β*1‐p.Arg85Cys colocalize with the plasma membrane marker, WGA (representative of five independent experiments). (E) Orthogonal views of a single z‐stack (YZ plane to right, XZ plane below) from immunofluorescence microscopy indicating colocalization between V5 and WGA signals.

Previous work with the *SCN1B*‐p.Cys121Trp variant, linked to GEFS+, showed normal cell surface expression in heterologous cells, but LOF in terms of I_Na_ modulation.^7^, ^20^ A biallelic *Scn1b*‐p.Cys121Trp mouse model showed a phenotype that is similar to DS.^21^ In contrast, heterologous expression experiments with the variant, *SCN1B‐*p.Arg125Cys, linked to a diagnosis of DS, showed that the mutant *β*1 protein was retained inside the cell and subsequently unavailable at the plasma membrane to modulate I_Na_.^15^ Growing the cells at a lower temperature, to permit proper protein folding, resulted in cell surface expression and normal I_Na_ modulation.^15^ To investigate the subcellular localization of this newly identified DEE‐linked *SCN1B* variant, p.Arg85Cys, which is located in the Ig loop domain (Fig. 7A and B),^23^ we performed cell surface biotinylation experiments comparing the polypeptide, *β*1‐p.Arg85Cys, to wild‐type (WT) *β*1 protein (Fig. 7A). Experiments were performed in Chinese hamster lung (CHL) fibroblasts that were stably transfected with soluble enhanced green fluorescent protein (eGFP), WT *β*1, or *β*1‐p.Arg85Cys. The WT *β*1 and *β*1‐p.Arg85Cys cDNA constructs each contained a C‐terminal, in‐frame, V5‐epitope tag followed by a 2A endoproteolytic sequence and eGFP on the 3’ end of the construct for detection of equimolar expression of a fluorescence marker protein. Total protein and neutravidin‐selected cell surface proteins were analyzed by western blot with anti‐V5 antibody. An antibody to HSP90 was used as an internal control to detect non‐cell surface proteins. Results shown in Figure 7C suggest that, similar to WT *β*1, *β*1‐p.Arg85Cys localizes to the cell surface. To confirm this result by another method, *β*1 subcellular localization in the same cell lines used in Figure 7B was assessed by confocal immunofluorescence microscopy. Cells were stained with fluorescently labeled wheat germ agglutinin (WGA), to identify the plasma membrane, and anti‐V5 antibody, to label WT or mutant *β*1 subunits. Colocalization between anti‐V5 and WGA was observed in both the WT *β*1 and *β*1‐p.Arg85Cys lines, confirming that the patient variant, β1‐p.Arg85Cys, can traffic to the plasma membrane (Fig. 7D). Orthogonal views of a single z‐stack from immunofluorescence microscopy are shown in Fig. 7E. No other overt differences in subcellular localization were observed between WT *β*1 and *β*1‐p.Arg85Cys.

### Electrophysiological analysis

**Figure 8:**
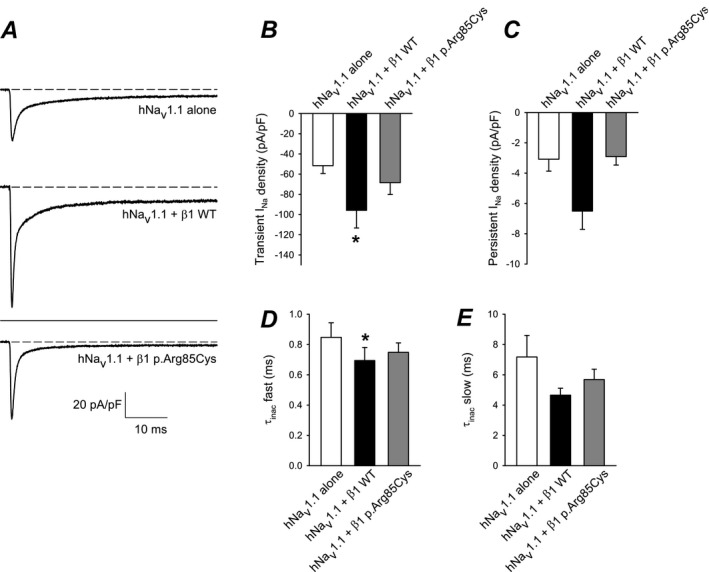
*β*1‐p.Arg85Cys does not modulate I_Na_ density or τ of inactivation. (A) Representative I_Na_ density traces; currents were evoked with a 50‐msec test pulse to 0 mv following a prepulse to − 120 mV. *Top:* Cells expressing hNa_v_1.1 alone (eGFP only). *Middle:* Cells expressing hNa_v_1.1 plus WT *β*1 subunits. *Lower:* Cells expressing hNa_v_1.1 plus *β*1‐p.Arg85Cys subunits. All traces are shown at the same scale. (B) Mean transient I_Na_ density measured at the peak from currents represented in (A). (C) Mean persistent I_Na_ density measured as the average current of the last 2 msec of the current to 0 mV represented in (A). (D and E) I_Na_ inactivation was fit to a double exponential equation and the mean *τ* for the fast and slow components were plotted. *n* = 13 cells per condition; *P < 0.05. Error bars indicate mean ± standard error of the mean. *P < 0.05. [Correction added on 06 December 2019 after first online publication: Figure 8 has been updated.]

**Figure 9:**
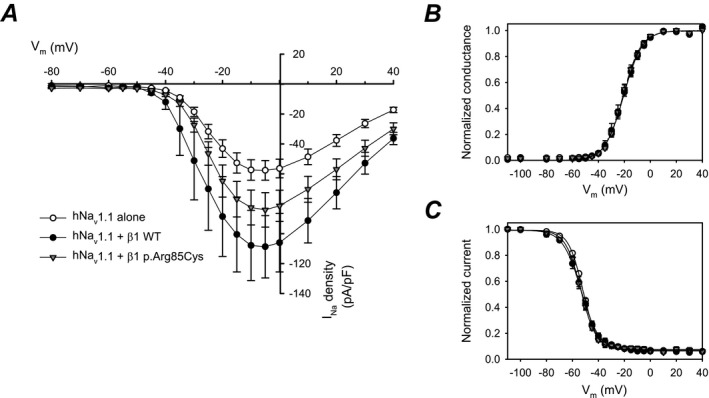
WT *β*1 subunit co‐expression increases hNa_v_1.1 I_Na_ density but neither WT *β*1 nor *β*1‐p.Arg85Cys affect the voltage dependence of hNa_v_1.1‐generated I_Na_. (A) Mean current‐voltage relationships of transient I_Na_: hNa_v_1.1 alone, tranfected with eGFP only, (*open circles, n* = *12 cells*), hNa_v_1.1 plus WT *β*1 (*closed circles, n* = *11 cells*), hNa_v_1.1 plus *β*1‐p.Arg85Cys (*triangles, n* = *13 cells*). (B) Activation curves from the data shown in (A). (C). Steady‐state inactivation curves recorded using a standard two‐pulse protocol (hNa_v_1.1, *n* = 12 cells; hNa_v_1.1 plus WT *β*1, *n* = 13 cells; hNa_v_1.1 plus *β*1‐p.Arg85Cys, *n* = 13 cells). [Correction added on 06 December 2019 after first online publication: Figure 9 has been updated.]

We next investigated the ability of *β*1‐p.Arg85Cys to modulate I_Na_. Human Na_v_1.1 channels (hNa_v_1.1) were chosen for this experiment due to their association with EIEE and DS.^24^ HEK‐hNa_v_1.1 cells were transiently transfected with WT *β*1, *β*1‐p.Arg85Cys, or eGFP for voltage‐clamp analysis. Representative I_Na_ density traces, showing transient and persistent currents, evoked by a 50‐msec test pulse to 0 mv following a prepulse to −120 mV are shown in Figure 8A and quantified in Figure 8B and C. WT *β*1 co‐expression with hNa_v_1.1 significantly increased transient and persistent I_Na_ density compared to hNa_v_1.1 alone. In contrast, co‐expression of *β*1‐p.Arg85Cys with hNa_v_1.1 did not significantly change these values. Figure 8B and E show quantification of the mean *τ* values for the fast and slow components, respectively, of I_Na_ inactivation. WT *β*1 significantly reduced the fast, but not slow component, while *β*1‐p.Arg85Cys had no significant effect on either value. Results shown in Figure 9 and Table 1 indicate that neither WT *β*1 nor *β*1‐p.Arg85Cys subunits affect the voltage dependence of hNa_v_1.1‐generated I_Na_.

**Table 1:** Voltage‐dependent properties. Data were obtained by fitting individual activation or inactivation curves to a Boltzmann equation.

	Voltage‐dependent properties						*n*
	Activation			Inactivation			
	*G*_max_ (nS)	*k* (mV)	*V*_1/2_ (mV)	*h* (mV)	*V*_1/2_ (mV)	*C*	
hNav1.1 alone (eGFP only)	1.07 ± 0.13	7.08 ± 0.40	−20.4 ± 0.8	−5.81 ± 0.41	−51.8 ± 0.4	0.07 ± 0.012	12
hNav1.1 + *β*1 WT	1.88 ± 0.41	6.72 ± 0.023	−20.8 ± 1.5	−6.90 ± 0.62	−53.4 ± 1.2	0.06 ± 0.008	13
hNav1.1 + *β*1‐p.R85C p.Arg85Cys	1.35 ± 0.16	6.73 ± 0.32	−20.7 ± 1.0	−5.69 ± 0.32	−53.6 ± 0.7	0.08 ± 0.079	13

## Methods

### Antibodies

Primary antibodies used were as follows: anti‐V5 (1:1000 dilution, Invitrogen), anti‐HSP90 (1:1,000 dilution, Enzo Life Science), or Wheat Germ Agglutinin Alexa Fluor^™^ 568 conjugate (1:200 dilution, ThermoFisher). Goat anti‐mouse HRP‐conjugated antibody was diluted to 1:10,000 (Invitrogen) for both V5 and HSP90 western blots. Alexa Fluor^™^ 647 (1:500 dilution) was used as a secondary antibody for anti‐V5 in immunocytochemistry experiments.

### Expression vectors

Human WT *β*1‐V5‐2AeGFP and *β*1‐p.Arg85Cys‐V5‐2AeGFP cDNAs were generated by gBLOCK from Integrated DNA Technologies. Both *β*1 constructs contained an in‐frame, C‐terminal V5‐epitope tag followed by a 2A endoproteolytic sequence and eGFP on the 3’ end of the construct. A control construct expressing eGFP only was amplified from the WT construct using gene‐specific primers and gel‐purified. cDNAs were inserted into pENTR‐SD/D TOPO Gateway entry vector by a TOPO reaction according to the manufacturer's instructions. All constructs were moved to the Gateway‐compatible destination vector, pcDNAdest40, using a LR clonase reaction according to the manufacturer's instructions.

### Cell lines

Chinese hamster lung fibroblasts were obtained from the American Type Culture Collection. A stable HEK cell line expressing human (h) Na_v_1.1 was a gift from GlaxoSmithKline. CHL and HEK‐hNa_v_1.1 cells were maintained at 37°C and 5% CO2 in Dulbecco's Modified Eagle Medium (DMEM) supplemented with 5% heat‐inactivated fetal bovine serum (Corning) and 100 U/mL penicillin/streptomycin (Gibco). Stably transfected CHL cells and HEK‐hNa_v_1.1 cells were supplemented with 600 *µ*g/mL G418 (Gibco). Stable *β*1‐V5‐2AeGFP or *β*1‐p.Arg85Cys‐V5‐2AeGFP cell lines were generated by transfecting 1 *µ*g of cDNA with 5 *µ*L of Lipofectamine 2000. About 48 h following transfection, cells were passaged into media containing 600 *µ*g/mL G418 and incubated until individual, eGFP‐positive colonies were visible (approximately 7–10 days). Clonal colonies were isolated, expanded, and verified for *β*1‐V5 or *β*1‐p.Arg85Cys‐V5 expression via western blot with anti‐V5 antibody. For electrophysiological analyses, HEK‐hNa_v_1.1 cells were transiently transfected with *β*1‐V5‐2AeGFP, *β*1‐p.Arg85Cys‐V5‐2AeGFP, or eGFP only (1 *µ*g of cDNA with 5 *µ*L of Lipofectamine 2000). Approximately 12 h following transfection, the cells were passaged at low density onto 35‐mm dishes. Cells were identified by eGFP fluorescence by an investigator blind to genotype.

### Cell surface biotinylation and western blot analyses

Stable cell lines were grown in 100‐mm tissue culture plates until 90–100% confluent. Cell surface proteins were biotinylated using the Cell Surface Protein Isolation Kit (Pierce) according to the manufacturer's protocol. Loading buffer containing 1% sodium dodecyl sulfate, 1 mmol/L *β*‐mercaptoethanol, and 0.2% dithiothreitol was added to samples and heated for 10 min at 85°C. Samples were separated on 10% Tris‐Glycine polyacrylamide gels, as noted in the figure legends, then transferred to nitrocellulose membrane (16 h, 55 mA, 4°C) and probed with antibodies as noted in the figure legends. Incubations with anti‐V5 and secondary antibody were completed using a SnapID (Millipore‐Sigma) with 10‐min incubations. Anti‐HSP90 was incubated overnight at 4°C in 2% milk in Tris‐buffered saline with 0.1% tween (TBST). Secondary antibodies to detect anti‐HSP90 were incubated at room temperature for 1 h. Immunoreactive bands were detected using West Femto chemiluminescent substrate (GE Health Sciences) and imaged on an iBrightFL1000 (Invitrogen).

### Immunocytochemistry and confocal microscopy

CHL cells stably overexpressing WT *β*1‐V5‐2AeGFP or *β*1p.Arg85Cys‐V5‐2AeGFP constructs, as indicated in the figure legend, were incubated with WGA for 10 min at 4°C. Cells were washed three times for 5 min each in Dulbecco's phosphate‐buffered saline (DPBS) at 4°C and immediately fixed with 4% paraformaldehyde at room temperature for 15 min. Following fixation, cells were quickly washed three times in DPBS and blocked in 90% DPBS, 10% goat serum, and 0.3% triton X‐100 for 1 h at room temperature in a dark, humidified chamber. Cells were incubated in anti‐V5 overnight in a dark, humidified chamber. Cells were washed three times for 10 min each with DPBS, incubated in secondary antibody (Alexa Fluor^™^ 647) for 2 h in a dark, humidified chamber at room temperature, and then washed three times for 10 min in DPBS. Slides were air dried and mounted in Prolong Gold with DAPI (Invitrogen). Stained cells were imaged on a Zeiss 880 confocal microscope at the University of Michigan Department of Pharmacology.

### Electrophysiological analysis

HEK‐hNa_v_1.1 cells were transiently transfected as described above. Electrophysiological recordings were performed ~12 h following final plating. Isolated I_Na_ was recorded using standard whole‐cell patch clamp, with previously described conditions.^25^ Briefly, isolated I_Na_ was recorded from GFP‐positive HEK cells at 21–22°C in the presence of a bath solution containing (in mmol/L): 120 NaCl, 1 BaCl_2_, 2 MgCl_2_, 0.2 CdCl_2,_ 1 CaCl_2_, 10 HEPES, 20 TEA‐Cl, and 10 glucose (pH = 7.35 with CsOH, osmolarity = 300–305 mosm). Fire‐polished patch pipettes were filled with an internal solution containing (in mmol/L): 1 NaCl, 150 N‐methyl‐d‐glucamine, 10 ethyleneglycoltetraacetic acid (EGTA), 2 MgCl_2_, 40 HEPES, and 25 phosphocreatine‐tris, 2 MgATP, 0.02 Na_2_GTP, 0.1 leupeptin (pH = 7.2 with H_2_SO_4_). All recordings were performed within 10 to 120 min after the culture medium was replaced by bath recording solution and the dish with cells was placed on the recording setup. Holding potential was −80 mV.

Voltage‐clamp analysis was performed using pClamp 10 (Molecular Devices) and SigmaPlot 11 (Systat software). Statistical analyses, *t*‐test, and one‐way ANOVA, were performed using SigmaPlot. Statistical significance was determined by *P* > 0.05.

## Discussion

Here, we provide the first detailed electroclinical investigation of an epilepsy patient with bilateral hearing loss presenting with a previously unreported, inherited homozygous *SCN1B* missense variant, p.Arg85Cys, suggesting that this genotype is linked to an early infantile DEE rather than typical DS. Moreover, a significant reduction of seizure severity and of the fever‐induced SE was observed after FFA add‐on that persisted after a 2‐year follow‐up. Functional analysis of *β*1‐p.Arg85Cys protein in heterologous cells showed loss of Na_v_1.1‐generated I_Na_ modulation in spite of normal levels of expression of the mutant protein at the cell surface. A previously described *SCN1B* LOF variant at this position, *β*1‐p.Arg85His, has been linked to atrial fibrillation familial type 13 in heterozygous patients.^26^ Similar to *β*1‐p.Arg85Cys shown here, *β*1‐p.Arg85His was found to be expressed at the cell surface but lost the ability to modulate Na_v_1.5‐generated I_Na_ when expressed in a heterologous system.^26^ Previous work has shown that *β*1‐mediated effects on current modulation are cell‐type specific.^5^ Additionally, the heterologous cells utilized in these studies are nonpolarized and do not form specialized membrane microdomains such as axon initial segments or intercalated discs. Future work that characterizes these patient variants in neurons or cardiomyocytes may provide additional insights into disease mechanisms.

Patients with homozygous *SCN1B* variants are very rare. Seven patients have been reported so far, all diagnosed with DS.^15^, ^16^, ^17^ However, a review of the literature suggests that six of these seven published cases do not strictly fit DS criteria. In DS, first seizures usually begin around 6 to 12 months of age, with prolonged febrile, generalized, clonic, or hemiclonic seizures, and a normal EEG background until 2 years of age. Development shows signs of decline after 2 years of age. Disease is progressively marked by additional seizures of multiple etiologies, ataxia, and pyramidal signs.^27^, ^28^

The first *SCN1B* DEE patient described by Patino et al. (*SCN1B*‐p.Arg125Cys)^15^ was similar to the present case but with some clinical characteristics in favor of DS, for example, the presence of refractory FS, myoclonus, and seizure triggers like vaccination, fever, and infection. Nevertheless, the disease evolution of the *SCN1B*‐p.Arg125Cys patient was worse than the usual DS course. The EEG was abnormal from 3 months of age, with central discharges. While the neonatal period was not reported, a tetrapyramidal syndrome with global hypotonia was noted at 13 months. The reported absence of SE was not typical of DS. The patient died due to aspiration pneumonia at 14 months. The second patient, described by Ogiwara et al. (*SCN1B*‐p.Ile106Phe),^17^ was more similar to DS. While initial seizures were not provoked by fever, fever‐sensitive seizures of various types, including myoclonic seizures, appeared around 6 months of age. Developmental decline occurred earlier than typical DS. The course of the disease was marked by a global developmental delay with progressive ataxia and intractable epilepsy. Interictal EEG was initially normal, but started to display multifocal spikes and slow waves, occurring singly or in bursts, around 1 year of age. In a less clinically detailed paper, Ramadan et al. reported five other cases of *SCN1B*‐linked EE from three consanguineous families (*SCN1B*‐p.Tyr119Asp and *SCN1B*‐c.449‐2Ala > Gly, which are likely splice site variants).^16^ No seizure triggers, like fever or vaccination, were mentioned. The ages of EEG completion and seizure types were not systematically specified. Epilepsy onset and developmental decline appeared early (1 or 2 months old) in four of the five cases. One of the three families had a different phenotype with microcephaly, dysmorphic features, and various malformations.

In the present patient case, development was already abnormal at birth. Epilepsy started early, at 3 months of age, with abundant EEG discharges. The course of the disease was severe with profound motor and cognitive delay. While the observed prolonged febrile myoclonic or hemiclonic seizures were more concordant with DS, electroclinical evidence suggests that *SCN1B*‐linked DEE is more severe, with an earlier onset of seizures and developmental delay. This idea is consistent with the negative result of the study of Kim et al, looking for *SCN1B* variants in a cohort of DS patients^29^ as well as with the finding of neuronal pathfinding deficits in *Scn1b* null mice at postnatal day 10 prior to seizure onset.^30^ Thus, we propose that the term, “early infantile DEE,” is more appropriate than EIEE, which is classically used in patients with a severe early onset epilepsy and profound developmental delay. The term “epileptic encephalopathy” refers to conditions where the epileptic abnormalities themselves are thought to contribute to the progressive disturbance of cerebral function, such that early effective intervention may improve developmental outcome.^31^ Whether the patient reported here, and more generally all patients with EIEE, fit this definition of epileptic encephalopathy is highly debatable because the neurological prognosis of these patients seems predominantly caused by preexisting brain dysfunction^31^ and not by the severity of the epilepsy. Our case illustrates this concept: development was already abnormal from birth, before the onset of epilepsy, and despite the resolution of febrile SE and significant improvement of seizure frequency with FFA add‐on, we did not observe a significant improvement in neurological development. Because neurological prognosis is predominantly caused by a developmental encephalopathy of genetic origin, early infantile DEE seems a more appropriate term than EIEE to describe the electroclinical picture of this patient.

The addition of FFA (0.6 mg/kg/d) to the anticonvulsant regimen of our patient resulted in a significant and prolonged reduction in seizure frequency with increased quality of life (Fig. 5), but no significant improvement in motor or cognitive development. FFA was developed originally as an appetite suppressant but was withdrawn from human use after reports of increased risk of cardiac valvulopathy and pulmonary hypertension in adult patients treated with high‐dose FFA for obesity.^32^ The potential utility of FFA in epilepsy emerged in the 1980s with its use in intractable self‐induced epilepsy^33^, ^34^ and in the 1990s with a remarkable effect on a group of patients who were shown later to present with *SCN1A‐*DS variants.^35^ The positive benefit/risk ratio profile of low‐dose FFA treatment in patients with DS was confirmed in a recently reported phase 3 study.^36^, ^37^ FFA antiepileptic activity is thought to arise via enhanced serotonergic neurotransmission by augmenting carrier‐mediated synaptic release of 5‐HT and by preventing reuptake.^38^, ^39^ In our patient, low‐dose FFA provided resolution of febrile SE and sustained, clinically meaningful, daily seizure reduction that persisted after a 2‐year follow‐up. FFA was well tolerated with no clinical and/or echocardiographic signs of cardiac valvulopathy or pulmonary hypertension. These results suggest that FFA may provide a promising treatment option in patients with homozygous *SCN1B*‐linked early infantile DEE.

Early infantile DEE resulting from homozygous *SCN1B* LOF variants may be similar to a new class of variants linked to a recurrent, missense *SCN1A* variant, p.Thr226Met, which is associated with a more severe clinical phenotype than typical DS. In *SCN1A*‐linked early infantile DEE, seizures and epileptic spasms are observed beginning at an average of 9 weeks of age, with concomitant development of hyperkinetic movement disorders, including choreoathetosis, dystonia, myoclonus, and perioral hyperkinesia. Patients have greater developmental impairments than DS, are nonambulatory, and the majority require feeding tubes.^40^ Heterologous expression of the mutant Na_v_1.1‐p.Thr226Met protein showed negative shifts in the voltage dependence of both activation and inactivation of I_Na_ compared to WT Na_v_1.1, which can be interpreted as both gain‐ and loss‐of‐function.^41^

To conclude, we present an eighth case of a homozygous variant in *SCN1B* with a phenotype of early infantile DEE, rather than DS, and a prolonged response to FFA. The pathogenic role of this variant has been assessed in vitro and, while FFA appears to be particularly efficient in resolution of febrile SE and reduction in seizure frequency in this patient, further studies are needed to identify new therapeutic options to improve the neurological development of this devastating disease. Importantly, a review of the literature in light of our results suggests that early infantile DEE is a more appropriate terminology than either EIEE or DS for *SCN1B*‐linked epileptic encephalopathy.

## Author contributions

AAB, JO, and BA performed and analyzed the molecular biology and cell biology experiments. LFL‐S performed and analyzed the electrophysiological experiments. LLI planned and supervised the molecular, cellular, and electrophysiological research and wrote the manuscript. AA and CS performed the clinical follow‐up and electroclinical description, prepared the figures, and wrote the manuscript. DL performed the gene panel. AS and BC performed FFA treatment and follow‐up. MV performed the ABR and generated the figure.

## Conflict of Interest

The authors declare no conflict of interest.
